# Use of Exposure Data to Establish Causality in Drug–Adverse Event Relationships: An Example with Desvenlafaxine

**DOI:** 10.3390/ph17010069

**Published:** 2024-01-03

**Authors:** Andrea Rodríguez-Lopez, Gina Mejía-Abril, Pablo Zubiaur, Sofía Calleja, Manuel Román, Francisco Abad-Santos, Dolores Ochoa

**Affiliations:** 1 Clinical Pharmacology Department, Hospital Universitario de La Princesa, Faculty of Medicine, Instituto de Investigación Sanitaria La Princesa (IP), Universidad Autónoma de Madrid (UAM), 28006 Madrid, Spain; arodriguezl.externo@salud.madrid.org (A.R.-L.); ginapaola.mejia@salud.madrid.org (G.M.-A.); sofiacalleja93@gmail.com (S.C.); manuel.roman@salud.madrid.org (M.R.); mdolores.ochoa@salud.madrid.org (D.O.); 2 Centro de Investigación Biomédica en Red de Enfermedades Hepáticas y Digestivas (CIBERehd), Instituto de Salud Carlos III, 28029 Madrid, Spain

**Keywords:** adverse event, adverse drug reactions, causality algorithms, safety, pharmacokinetics

## Abstract

Causality algorithms help establish relationships between drug use and adverse event (AE) occurrence. High drug exposure leads to a higher likelihood of an AE being classified as an adverse drug reaction (ADR). However, there is a knowledge gap regarding what concentrations are predictive of ADRs, as this has not been systematically studied. In this work, the Spanish Pharmacovigilance System (SEFV) algorithm was used to define the relationship between the AE occurrence and drug administration in 178 healthy volunteers participating in five desvenlafaxine single-dose clinical trials, a selective serotonin and norepinephrine reuptake inhibitor that may cause dizziness, headache, nausea, dry mouth, constipation and hyperhidrosis. Eighty-three subjects presented 172 AEs that were classified as possible (101), conditional (31), unrelated (24) and probable (16). AUC_∞_ and C_max_ were significantly higher in volunteers with vs. without ADRs (5981.24 ng·h/mL and 239.06 ng/mL and 4770.84 ng·h/mL and 200.69 ng/mL, respectively). Six of 19 subjects with conditional AEs with an SEFV score of 3 points presented an AUC_∞_ ≥ 6500 ng·h/mL or a C_max_ ≥ 300 ng/mL (i.e., above percentile 75) and were summed one point on their SEFV score and classified as “possible” (4 points), improving the capacity of ADR detection.

## 1. Introduction

Adverse events (AEs) are defined as any undesirable event experienced by the subject during the administration of the drug, whether or not related to the drug. Adverse drug reactions (ADRs) are defined as any noxious and unintended response to a drug, thus including those derived from any use, abuse and medication errors [1]. They constitute a very important cause of morbimortality worldwide, occur in 10% of outpatients and cause 5–10% of hospital admissions, with 8.4% in a study recently conducted in the Hospital Universitario de La Princesa, Madrid (Spain) [2]. In addition, they are suffered by 10–20% of hospitalized patients, which increases their average length of hospital stay. In Spain, the incidence of hospitalized patients dying from ADRs is 7% [3].

Pharmacovigilance (PV) is the pharmacological science concerned with the collection, detection, evaluation, monitoring and prevention of adverse reactions to medicines. The appropriate and effective monitoring of ADRs, i.e., pharmacovigilance, is the best way to protect public health [4]. Causality assessment is the evaluation of the likelihood that a particular treatment is the cause of an observed adverse event. It assesses the relationship between a drug treatment and the occurrence of an adverse event. It is an important component of pharmacovigilance, contributing to a better assessment of the benefit–risk profile of medicines [5] and is an essential part of the evaluation of ADR reports in early warning systems and for regulatory purposes. Numerous methods for the causality assessment of adverse drug reactions (ADRs) have been published, such as the Jones’ algorithm [6], the Naranjo algorithm [7], the Yale algorithm [8], the Bégaud algorithm [9] or the SEFV algorithm [10], which is a modification of the one published by Karch and Lasagna [11]. Algorithms, which are structured systems specifically designed to identify an ADR, should theoretically provide a more objective determination of causality. However, there are also problems associated with algorithms; for instance, the answers to some of the questions in the algorithm may be influenced by clinical judgment, so specific training is required; furthermore, the questions in algorithms are often arbitrarily weighted; moreover, some questions include YES/NO answer options, which may not be fully appropriate, as some uncertainty may not be captured with these answers [12].

In this paper, we have focused on the SEFV algorithms [10], which consider chronology, prior knowledge, withdrawal effect, re-exposure and the presence of an alternative cause, which may be used to establish causality. The final score obtained from the algorithm classifies the causal relationship as definite, probable, possible, conditional or unrelated. The first three are considered ADRs [10]. The SEFV algorithm contemplates drug concentrations at the time of the reaction, but this parameter is not usually assessed as it is rarely available in clinical practice. Furthermore, if available, there is a lack of consensus on what exposures can be considered toxic for many drugs. Conditional AEs can be the most difficult to classify. Knowing which individuals were exposed to a toxic concentration of the drug will help in identifying ADRs in this group; not knowing the toxicity thresholds of a drug may lead to the underreporting of ADRs and to a worse characterization of drug safety profiles.

This is an extension of a previous study [13], where the impact of genetic variation of desvenlafaxine exposure and safety was investigated. Although no genetic polymorphism was related to pharmacokinetic variability or ADR incidence, a clear exposure–safety relationship was observed. Desvenlafaxine is a selective serotonin and norepinephrine reuptake inhibitor (SSRI). It is used to treat psychiatric conditions including major depressive disorder, generalized anxiety disorder, social anxiety disorder and panic disorder. The recommended starting dose of desvenlafaxine is 50 mg once daily, with a therapeutic range of 50–200 mg once daily [14]; 50 mg/day has been shown to be an effective dose [15]. Although doses up to 400 mg per day have been tested, there is no evidence that doses higher than 50 mg/day are more effective [16]. Therefore, the lowest effective dose should be maintained due to the risk of dose-related ADR. Treatment with desvenlafaxine 50 and 100 mg/day is generally safe and well tolerated, but it is not fully exempt from ADR occurrence, which can lead to problems with treatment adherence and, consequently, effectiveness [14]. Therefore, we decided to increase the sample size and investigate if drug exposure could be helpful for the identification of ADRs in individuals with AEs conditionally related to drug intake. Moreover, we aimed to further evaluate the impact of sex, biogeographic origin, dose and feeding conditions on desvenlafaxine pharmacokinetic parameters and on the occurrence of ADRs.

## 2. Results

### 2.1. Pharmacokinetics

Of the 180 volunteers who participated in the five clinical trials, 178 (82 women and 96 men) were included in the pharmacokinetic analysis and 2 were excluded due to the non-completion of the clinical trial and the lack of pharmacokinetic data. However, 6 subjects did not complete the second period per voluntary withdrawal. In addition, AUC_∞_ could not be calculated in 3 subjects, which is explained by the limitations of non-compartmental pharmacokinetic analyses, where the AUC from t to infinite is estimated as −Ct/k. In these 3 subjects, the k value was close to zero (or even positive), which does not allow a correct estimation of the extrapolated AUC and therefore the AUC_∞_.

Women presented lower height and weight than men (*p* < 0.001) (Table 1). Europeans were younger than Latin Americans or Sub-Saharan Africans (*p* < 0.050) (as only one volunteer self-identified as Sub-Saharan African, he was included in the Latin Americans group, named ‘Other’) and exhibited greater height and lower body mass index (BMI) (*p* < 0.050, *p* < 0.001, respectively) (Table 1). No differences were found between the clinical trials in terms of age, height, weight or BMI (Table 1).

For desvenlafaxine 50 mg, median (Q1–Q3) AUC_∞_ and C_max_ were 3088.95 (2701.86–3695.88) ng·h/mL and 135.21 (104.34–169.67) ng/mL, respectively, and 6305.37 (5369.96–7231.08) ng·h/mL and 275.35 (215.78–339.04) ng/mL for the 100 mg dose, showing linear pharmacokinetics. AUC_∞_ and AUC_∞_/D were significantly higher in women compared to men (*p* < 0.001) but not AUC_∞_/DW. C_max_, C_max_/D and C_max_/DW were significantly higher in women compared to men (*p* < 0.001; *p* < 0.001; *p_uv_* < 0.001, *p_mv_* < 0.001, β = 21.94, R^2^ = 0.465, respectively) (Table 2). AUC_∞_/D was significantly higher in Europeans compared to volunteers with other biogeographic origins (*p* = 0.044); however, C_max_/DW was significantly higher in volunteers with other biogeographic origins compared to Europeans (*p* = 0.025) (Table 2) but not C_max_ or C_max_/D. AUC_∞_/DW was significantly higher in fed volunteers compared to fasting volunteers (*p_uv_* < 0.001, *p_mv_* < 0.001, β = 369.87, R2 = 0.038). C_max_, C_max_/D and C_max_/DW were significantly higher in fed compared to fasting volunteers (*p* < 0.001; *p* < 0.001; *p_uv_* < 0.001 *p_mv_* < 0.001, β = 76.76, R^2^ = 0.465, respectively) (Table 2).

### 2.2. Safety

There were 172 AEs in 83 subjects, and 77 of 178 healthy volunteers (350 exposures) experienced at least one ADR, for a total of 117 ADRs. The observed ADRs were classified into the following System Organ Class (SOC): gastrointestinal disorders (*n* = 46), nervous system disorders (*n* = 59), general disorders and administration site conditions (*n* = 4), metabolism and nutrition disorders (*n* = 4), cardiac disorders (*n* = 2), psychiatric disorders (*n* = 1) and vascular disorders (*n* = 1). AUC_∞_ was significantly higher in volunteers with ADRs (5981.24 [4055.86–7209.16] ng·h/mL) compared to volunteers without ADRs (4770.84 [3138.69–6375.76] ng·h/mL) (*p* = 0.002). C_max_ was likewise significantly higher in volunteers with ADRs (239.06 [181.96–300.67] ng/mL) compared to volunteers without ADRs (200.69 [138.83–295.04] ng/mL) (*p* = 0.041).

A total of 28.9% of women had ADRs compared to 16.8% of men, (*p_uv_* = 0.007, *p_mv_* < 0.001, OR = 2.53). A total of 27% of subjects receiving 100 mg had ADRs compared to 15.1% of those receiving 50 mg (*p_uv_* = 0.009, *p_mv_* = 0.001, OR = 2.37). AUC_∞_ and C_max_ were significantly higher in volunteers with gastrointestinal disorders compared to volunteers without gastrointestinal disorders (6481.22 [5473.62–7573.20] ng·h/mL and 4751.46 [3146.87–6375.76] ng·h/mL, respectively, *p* = 0.001; 269.89 [209.31–343.47] ng/mL and 200.07 (138.93–290.629) ng/mL, respectively, *p* = 0.001) but not in those with/without nervous system ADRs. The remaining ADRs occurred in 4 subjects or less and were thus not analyzed.

**Figure 1:**
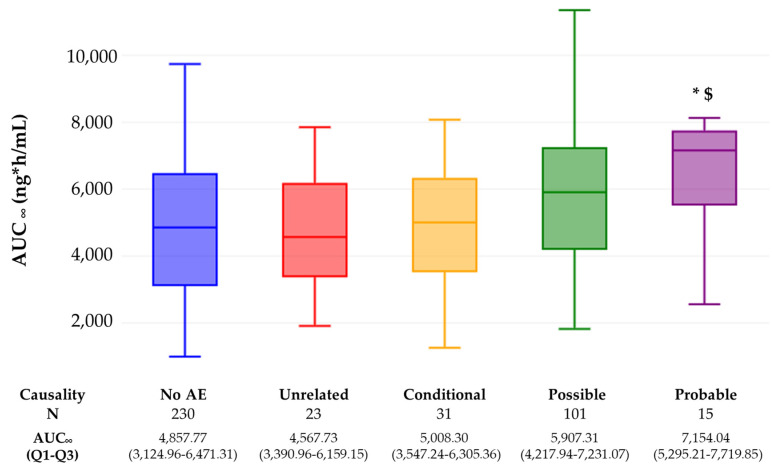
AUC_∞_ values as a function of AE causality. * *p* < 0.05 compared to no AE, ^$^ *p* < 0.05 compared to unrelated. N: number of exposures (no AE) or number of unrelated, conditional, possible or probable AEs.

AUC_∞_ was significantly lower in volunteers who had no AEs compared to volunteers who had ADRs with possible causality (*p* = 0.003) and probable causality (*p* = 0.035), as well as in volunteers who had unrelated AEs compared to volunteers who had ADRs with probable causality (*p* = 0.048) (Figure 1). No significant differences were observed for C_max_.

AUC_∞_ and C*_max_* values tended to be higher in volunteers who presented Aes conditionally related to drug intake, with a score of 3 points compared to those with 1 or 2 points (Table 3).

Of a total of 19 AEs with conditional causality and 3 points, 6 corresponded to subjects with AUC_∞_ and/or C_max_ values above the percentile 75 of all subjects (AUC_∞_ = 6549.21 ng·h/mL and C_max_ = 296.42 ng/mL); remarkably, that AUC_∞_ value lies between the median AUC_∞_ values of possible or probable ADRs, and the C_max_ value lies above the median value of probable ADRs. Therefore, it was justified to recalculate the score for these AEs via summing a point that corresponds to question 7 of the SEFV causality algorithm, regarding the evidence of toxic exposure. Thus, 6 of the 19 conditional AEs with an initial score of 3 points became ADRs with possible causality (i.e., 4 on the SEFV algorithm) (Table 4). These symptoms (dizziness, headache, nausea, vomiting, decreased appetite and muscle spasms) are typically caused by desvenlafaxine.

## 3. Discussion

In this study, we focused on the SEFV algorithm and on how to reduce this subjectivity in one of its questions, specifically number 7 “Investigations”, which considers adding a point if there is evidence of toxic exposure to a drug. Here, we observed that desvenlafaxine AUC_∞_ and C_max_ values of approximately 6500 ng·h/mL and 300 ng/mL, respectively, or higher, can be considered toxic, and an additional point could be summed in patients presenting such. To the best of our knowledge, scarce literature supports the therapeutic drug monitoring (TDM) of desvenlafaxine. However, TDM is frequent for venlafaxine, where dose/concentration dependency is observed towards drug effectiveness and safety, with a therapeutic range of 140 to 600 ng/mL for venlafaxine + desvenlafaxine concentrations (trough levels) and a 144 to 302 ng/mL range for desvenlafaxine concentrations [17]. Interestingly, the upper limit of the therapeutic range previously reported pretty well matches the C_max_ threshold value considered “toxic”. The fact that our study is a single dose implies that the steady-state C_max_ will be higher, but also that some ADRs will disappear after a few weeks of treatment. However, this threshold and the data available in the literature confirm that, if a trough level above 300 ng/mL is observed in routine clinical practice, a point can be confidently added on the SEFV scale.

Desvenlafaxine is a metabolite of venlafaxine, so the exposure data observed in this study would also apply to venlafaxine, albeit partially, as both venlafaxine and desvenlafaxine have serotonin and norepinephrine reuptake inhibitory properties [18]. ADRs triggered by serotonin or norepinephrine disposition at the synapse may be shared, but the affinities for the two transporters are different; furthermore, venlafaxine, but not desvenlafaxine, is reported to be a dopamine reuptake inhibitor [18], which may lead to the occurrence of different ADRs not observed with desvenlafaxine treatment. Also, this study could be carried out with other drugs, especially those that have been shown to have a dose-dependent relationship with the occurrence of ADRs, but the methodology is valid for any drug.

Desvenlafaxine pharmacokinetic parameters were consistent with the information available in the literature [14] and our previous work [13], where linear pharmacokinetics was observed. Here, healthy female volunteers presented a higher C_max_/DW; this association was also described in our previous work with the same clinical trials but fewer volunteers [13] and may be explained by sex-specific physiological differences in drug absorption [19]. Fed healthy volunteers showed a higher AUC_∞_/DW and C_max_/DW compared to fasting healthy volunteers; this effect in C_max_ is well described in previous studies [20], including ours [13], where only a tendency was observed for AUC_∞_/DW, consistent with the present study. Overall, the use of DW correction seems to reduce pharmacokinetic variability and increase statistical power, allowing associations with feeding conditions and sex to be established in the multivariate analysis, whereas in other models where weight is a confounding factor, these associations are not observed.

When focusing on ADRs, women showed a higher incidence of ADRs compared to men, which is mainly explained by the difference in body weight between the sexes, which explains the higher values of DW-uncorrected pharmacokinetic variables in females. Furthermore, volunteers receiving desvenlafaxine 100 mg showed a higher incidence of ADRs compared to those receiving desvenlafaxine 50 mg. Furthermore, volunteers with gastrointestinal disorders showed a significantly higher desvenlafaxine exposure compared to volunteers without such ADRs. This suggests that weight-informed dose adjustments may be necessary to control drug exposure and minimize the risk of ADRs.

A systematic review and network meta-analysis based on 522 double-blind trials involving 116,477 patients randomized and 21 different first- and second-generation antidepressants or placebo found that desvenlafaxine had lower efficacy and acceptability than other antidepressants, such as amitriptyline, escitalopram, mirtazapine, paroxetine or venlafaxine [21]. This may suggest that more research is needed with desvenlafaxine to clearly define the therapeutic range and reduce the likelihood of ADRs.

A significant limitation in current practice is the unavailability or inconsistency in assessing drug concentrations at the time of AEs. Nonetheless, our work supports the usefulness of drug exposure measurements to establish causality relationships. We recommend considering drug levels when available in AE-causality assessments; we encourage practitioners to request the determination of drug plasma levels in the event of AEs.

While the results of this work may be valuable, they should be validated in larger, independent and more diverse populations. However, we believe it is important to also consider the virtues of our model, based on bioequivalence clinical trials, which allow us to analyze the association very clearly and precisely, without confounding factors, where all the AEs are collected. This would not be possible under clinical practice conditions with patients.

## 4. Materials and Methods

### 4.1. Study Population and Study Design

This is a retrospective observational study based on five single-dose bioequivalence trials (A, B, C, D, E) of desvenlafaxine conducted between 2019 and 2021 at the Clinical Trials Unit of the Hospital Universitario de La Princesa (UECHUP), Madrid (Spain) (Table 5). In three of them (A, D, E), two desvenlafaxine 100 mg prolonged-release tablet formulations were administered once; in two of them (B, C), two desvenlafaxine 50 mg prolonged-release tablet formulations were administered. All of them were open-label, crossover and randomized clinical trials, with two sequences, two periods and a wash-out period of at least 7 days. In each period, volunteers were hospitalized from 10 h before to 24 h after dosing in both periods. The formulations were administered orally under fasting (A, B, D) or fed (C, E) conditions. In fed conditions, the subjects were given a high-fat and high-calorie breakfast, according to European Medicines Agency (EMA) guidelines [22], consisting of a fried egg (80 g), fried potatoes (100 g), a sausage (50 g), a slice of bread (60 g) and a glass of milk (200 mL), accompanied by a glass of water (200 mL), within a period of 30 min prior to drug administration.

During hospital admission and at additional visits in each period, 21 blood samples were collected from pre-dose to 72 h after drug administration. Drug concentration determinations were outsourced to an external laboratory. The analytical method was based on high-performance liquid chromatography coupled with tandem mass spectrometry (HPLC-MS/MS), with a lower limit of quantification (LLOQ) of 1 ng/mL, validated according to EMA guidelines [23].

Information on demographic parameters (age, sex, biogeographic origin, weight, height, BMI), pharmacokinetics and occurrence of AEs and ADRs was collected from clinical trials reports.

The inclusion criteria for participation in the bioequivalence trials included healthy men or women between 18 and 55 years. The exclusion criteria included: any organic or psychiatric pathology, use of any pharmacological treatment in the previous 48 h, BMI outside the range of 18.5–30 kg/m^2^, history of any type of hypersensitivity to drugs, positive detection of drugs of abuse, smokers, alcoholics or alcohol intoxication in the previous week, having donated blood in the previous month, pregnancy or breastfeeding, having participated in a similar study in the previous 3 months, inability to follow instructions or to collaborate during this study and history of difficulty swallowing.

The Independent Ethics Board of the Hospital Universitario de La Princesa approved this study on 23 of November, 2021 (registry number 4627). Due to the observational and retrospective nature of this study, and having already collected informed consent from the healthy volunteers for the bioequivalence trials, the request for an additional informed consent was waived. The Good Clinical Practice guidelines [24], the Spanish and European Biomedical laws and the principles of the Declaration of Helsinki were respected [25].

### 4.2. Pharmacokinetic Parameters

Pharmacokinetic analysis was performed using the professional version of Phoenix WinNonlin (Scientific Consulting, Inc, Cary, NC, USA). The AUC infinite (AUC_∞_) resulting from the sum of two partial AUCs was used: (a) AUC_0-t_ between the initial and last detectable concentrations calculated using the trapezoidal method; and (b) AUC_t-∞_, calculated as C/k, where C is the last detectable concentration and k is the slope of the line obtained by linear regression from the points corresponding to the elimination phase of the drug. AUC_∞_ could not be calculated for all subjects because there was not a clear elimination phase. The C_max_ was obtained directly from plasma concentration data.

Pharmacokinetic data of the study drug were obtained for each subject. Each subject received two doses of the drug; thus, two exposures were counted for each subject. Subjects participating in more than one clinical trial were considered as independent subjects.

### 4.3. Safety

All AEs spontaneously reported by volunteers or reported in response to an open question were recorded. AEs were coded using MedDRA terminology [26], assigned a preferred term (PT) and grouped according to the SOC. The causality assessment was conducted by clinical pharmacologists with specific training in clinical trials and pharmacovigilance. The SEFV algorithm was used for evaluation of causality and consists of seven questions. Temporal sequence, prior knowledge, withdrawal effect, re-exposure and alternative cause are five questions that can have different answers, with a score ranging from −3 to +3 points. The remaining two questions, which are the contributing factors favoring the causal relationship and the complementary investigations (i.e., plasma drug concentrations), can be answered yes or no, with a score of 1 or 0, respectively. The score obtained with the algorithm classifies the causal relationship into five categories: ≤0 unrelated, 1–3 conditional, 4–5 possible, 6–7 probable and 8 definite [10]. Only AEs with a definite, probable or possible causality were considered ADRs.

### 4.4. Statistical Analysis

The IBM SPSS Statistics (version 23, SPSS Inc, Chicago, IL, USA) was used for statistical analysis. AUC∞ and C_max_ were divided by the dose (D) and by the dose/weight ratio (DW) to correct dose or dose and weight effects, respectively, on bioavailability. The six variables (AUC_∞_, AUC_∞_/D, AUC_∞_/DW, C_max_, C_max_/D and C_max_/DW) were analyzed to determine which statistical model is superior to control for pharmacokinetic variability related to sex-related weight differences. The Shapiro–Wilk test was used to check variable distributions. A logarithmic transformation was applied and normality was re-analyzed to ensure normal distribution of the log-transformed variables. In the univariate analysis, pharmacokinetic parameters were analyzed according to sex, self-reported biogeographical origin, feeding conditions and ADR presence. ANOVA or *t*-tests were used to compare means for variables with a normal distribution. For variables that were not normally distributed, non-parametric tests were used: a Mann–Whitney or a Kruskal–Wallis test. Multivariate analyses were performed on the DW-corrected variables using linear regression, including those independent variables that were significantly associated with the dependent variable in the univariate analysis (i.e., with univariate *p*-values (*p_uv_*) lower than 0.05). The multivariate *p*-value (*p_mv_*), non-standardized β-coefficient (β) and R^2^ are presented for significant associations.

The incidence of ADRs was analyzed using Fisher exact tests or chi-squared tests, when appropriate, according to sex, biogeographical origin and feeding conditions.

## 5. Conclusions

Knowledge of the drug concentrations of desvenlafaxine can help improve the evaluation of the causality of adverse events. Desvenlafaxine AUC_∞_ and C_max_ values of approximately 6500 ng·h/mL and 300 ng/mL, respectively, or higher, can be considered toxic and an additional point in the SEFV causality algorithm could be summed in patients presenting such. If TDM values are available, trough levels above 300 ng/mL can similarly be considered toxic and a point may be summed. The weight-based prescription of desvenlafaxine may be appropriate to control for drug exposure and risk for ADR occurrence.

## Figures and Tables

**Table 1:** Demographic characteristics of volunteers included in the study.

Variable		N	Age (Years)	Height (m)	Weight (kg)	BMI (kg/m^2^)
			Median (Q1–Q3)	Median (Q1–Q3)	Median (Q1–Q3)	Mean (SD)
Sex	Male	96	29 (25–34)	1.75 (1.70–1.80)	75.80 (66.75–83.98)	24.68 (2.77)
	Female	82	29 (24–36)	1.61 (1.60–1.67) *	62.30 (57.45–67.53) *	23.90 (2.53)
Biogeographic origin	European	51	28 (22–33) ^$^	1.72 (1.66–1.8) ^$^	65.50 (59.50–74.80)	23.05 (2.85) *
	Other ^#^	127	30 (25–36)	1.70 (1.6–1.75)	69.70 (62.60–78.20)	24.84 (2.44)
Clinical trial	A	36	28 (24–34)	1.70 (1.62–1.76)	67.40 (63.50–74.33)	23.86 (2.51)
	B	36	29 (25–33)	1.70 (1.60–1.70)	67.55 (62.30–77.80)	24.25 (2.34)
	C	34	31.5 (27–38)	1.70 (1.70–1.80)	68.70 (61.00–82.38)	24.34 (2.41)
	D	36	28.5 (25–36)	1.70 (1.61–1.77)	66.85 (61.00–76.75)	24.32 (2.88)
	E	36	28.5 (22–36)	1.70 (1.61–1.75)	68.75 (60.00–82.00)	24.88 (3.23)
Total (Mean (SD))		178	30.60 (8.07)	1.70 (0.09)	70.17 (11.66)	24.33 (2.69)

N: number of volunteers * *p* < 0.001, ^$^ *p* < 0.05, ^#^ Other: Latin Americans (126) + Sub-Saharan African (1).

**Table 2:** Pharmacokinetic parameters according to sex, biogeographical origin and fed conditions.

Variable		AUC_∞_ (ng·h/mL)		AUC_∞_/D (ng·h/mL*mg)		AUC_∞_/DW (kg*ng·h/mL*mg)		C_max_ (ng/mL)		C_max_/D (ng/mL*mg)		C_max_/DW (kg*ng/mL*mg)	
		N	Median (Q1–Q3)	N	Median (Q1–Q3)	N	Median (Q1–Q3)	N	Median (Q1–Q3)	N	Median (Q1–Q3)	N	Median (Q1–Q3)
Sex	Male	190	4601.77 (2981.92–5988.57)	190	57.95 (49.56–65.32)	190	4361.7 (3821.39–4807.32)	191	179.62 (131.35–248.41)	191	2.34 (1.94–2.92)	191	178.06 (147.91–222.31)
	Female	157	6136.57 (3998.87–7254.42) *	157	69.75 (61.89–79.35) *	157	4374.77 (3871.71–4869.57)	159	257.39 (186.18–343.11) *	159	3.21 (2.53–3.85) *	159	201.45 (161.33–237.33) *
Biogeographic origin	European	99	5829.78 (4212.25–7032.24)	99	65.07 (56.92–78.06) ^$^	99	4350.45 (3821.05–4981.42)	101	214.54 (172.41–396.81)	101	2.66 (2.03–3.39)	101	168.15 (140.84–223.85)
	Other	248	4770.84 (3094.42–6378.03)	248	61.95 (52.68–71.79)	248	4373.02 (3846.55–4810.85)	249	210.54 (136.53–297.74)	249	2.76 (2.16–3.39)	249	188.18 (156.19–233.56) ^$^
Feeding conditions	Fasting	208	5297.56 (3223.13–6752.41)	208	61.61 (52.7–71.51)	208	4300.33 (3737.77–4702.98)	211	198.94 (123.2–270.45)	211	2.32 (1.97–2.87)	211	160.95 (140.17–183.72)
	Fed	139	4415.57 (3390.96–6356.21)	139	64.5 (54.85–75.22)	139	4575.56 (4040.28–5052.86) *	139	244.42 (165.75–339.04) *	139	3.32 (2.78–4.02) *	139	233.88 (208.16–264.09) *
Total		347	4956.22 (3319.66–6549.21)	347	62.68 (53.83–72.61)	347	4371.66 (3831.29–4834.93)	350	212.29 (146.39–296.42)	350	2.73 (2.13–3.39)	350	185.75 (154.32–231.69)

N: number of exposures. AUC_∞_/D: dose-corrected area under the curve. C_max_/D: dose-corrected maximum plasmatic concentration. AUC_∞_/DW: dose-weight-corrected area under the curve. C_max_/DW: dose-weight-corrected maximum plasmatic concentration. * *p* < 0.001, ^$^ *p* < 0.05, underlined: multivariate *p*-value (*p_mv_*) < 0.001.

**Table 3:** Pharmacokinetic parameters in volunteers with conditional AEs.

SEFV Score	N	AUC_∞_ (ng·h/mL)	C_max_ (ng/mL)
		Median (Q1–Q3)	Median (Q1–Q3)
1 or 2	12	4344.94 (3471.56–6177.11)	177.19 (138.11–233.21)
3	19	5046.77 (3620.79–6385.53)	243.52 (175.60–330.11)
Total	31	5008.30 (3547.24–6305.36)	201.06 (147.89–279.71)

**Table 4:** Conditional adverse events, pharmacokinetic parameters and SEFV algorithm causality score.

MedDRA SOC	MedDRA PT	AUC_∞_ (ng·h/mL)	C_max_ (ng/mL)	Initial Score	New Score
Nervous system disorders	Headache	5792.34	178.66	3	3
	Headache	2138.86	67.39	3	3
	Presyncope	3620.8	208.05	3	3
	Headache	6305.37	243.52	3	3
	Dizziness	7514.45 *	455.74 *	3	4
	Headache	6284.71	330.11 *	3	4
	Headache	4705.14	200.32	3	3
	Psychomotor hyperactivity	6481.22	179.62	3	3
	Presyncope	5008.3	279.71	3	3
	Headache	5008.3	279.71	3	3
	Headache	4597.18	256.68	3	3
Gastrointestinal disorders	Dry mouth	3547.24	175.6	3	3
	Vomiting	6385.53	330.58 *	3	4
	Nausea	7440.14 *	380.9 *	3	4
Respiratory, thoracic and mediastinal disorders	Epistaxis	3099.63	116.37	3	3
Metabolism and nutrition disorders	Decreased appetite	6284.71	330.11 *	3	4
General disorders and administration site conditions	Asthenia	5046.78	147.89	3	3
Eye disorders	Vision blurred	1258.01	67.78	3	3
Musculoskeletal and connective tissue disorders	Muscle spasms	7521.95 *	357.21 *	3	4

* Values above the percentile 75 of all subjects.

**Table 5:** Characteristics of the clinical trials included in this study.

Internal Code	EudraCT Code	Dose	Type of Study	Sample Size
A	2019-000628-17	100 mg	Single dose—fasting	36
B	2019-002739-26	50 mg	Single dose—fasting	36
C	2019-004289-16	50 mg	Single dose—fed	36
D	2019-004882-41	100 mg	Single dose—fasting	36
E	2020-003002-31	100 mg	Single dose—fed	36
			Total	180

## Data Availability

Data belong to the clinical trials’ sponsors and may be accessible upon reasonable request to the corresponding authors.
